# Poisoned Sausages

**Published:** 1857-04

**Authors:** 


### ARTICLE VIII.

### *Poisoned Sausages.*

It was towards the end of the eighteenth century that the attention of physicians appears to have been first directed to the peculiar pathological symptoms resulting from a poisoning of an acute or chronic character, induced by the eating of sausages and other kinds of stale animal food. The poisonous effects have been especially observed after eating such kinds of animal substances which, to all appearance, had undergone but little change, or which, at least, had no stronger or more unpleasant smell, or discoloration, or other more decided indications of decomposition than what is termed ripe game, which is so often and freely partaken of without any bad effects resulting.

It is a curious circumstance, that, as appears to have been invariably the case, the peculiar poisonous effects should result only from the eating of animal substances that have been subjected to a more or less prolonged cooking, and hence blood and liver puddings, especially those that have been smoked, have been found very often to produce a poisonous effect on such as partake of them

Although the particular change in the food upon which the development of its poisonous properties is produced is not fully understood, it has been attempted to be explained hypothetically. Thus Kerner supposed that a peculiar acid, developed by the fatty matter of the food, was the poisonous agent, but Buchner subsequently showed that the only acid found in poisonous sausages is the innocuous acetic, and that the poisonous substance upon which their noxious qualities depend, named by him pyroadeptic ether, was more similar to the etherial oils than to an acid. Since then, it has been found to be analogous to the volatile fatty oils.

The greatest number of instances of sausage poisoning have been observed at Wurtemberg. But the most remarkable instance on record is that which occurred at Andelfingen, in the Swiss, January 10, 1839, when, of six hundred persons congregated on the occasion of a musical festival, who had dined together in one tent, 444 were taken sick, and nine died. Of those who escaped, some had eaten none of the unwholesome food; others but a very small quantity, which was almost immediately rejected by vomiting. It is worthy of remark, that the typhoid form of disease produced, on this occasion, by eating of the poisonous viands, subsequently assumed a contagious character.

In the *Med. Correspondenzblatt*, of the Association of Wurtemberg Physicians, vol. xxviii, Nos. 41 and 42, Dr. Berg, chief physician to the poor in Langenberg, gives a detailed history of poisonous effects resulting from eating unwholesome sausages, that occurred in the kingdom of Wurtemberg, April, 1855, and which, in three cases, terminated fatally. He states that J. G. M., a peasant of Oberstetten, made a number of blood and liver puddings out of the lungs and liver and two pitchers of the blood of an ox, purchased the preceding day, and the similar parts of a hog that had been killed on the seventh of April, and with a portion of the same material of which these were composed, he filled the stomach of the hog. These things were all completed on the same day, and hung up in the chimney to be smoked, from whence they were taken as they were wanted for the use of the family. On the 19th of April, the last of the liver puddings were eaten, with potatoes, by five grown persons and three children. Each of the former ate half a pudding, except the peasant M., who ate a whole one at dinner, and in the afternoon and at night another. The same persons partook, at noon on the following day, of sourcrout, peas, and of the sausage mixture with which the hog's stomach was filled, notwithstanding all the members of the family had been sick the preceding day. Of these, three died.

"1. A shepherd, aged 28 years, robust, and otherwise in good health. He had complained, as early as the 17th of April, of lassitude and cutting pains in the stomach. On the evening of the 19th, he experienced intense thirst, pains in the stomach and loss of appetite. The next morning whilst in the field, he was attacked with severe pain of the stomach, and frequent vomiting; he, nevertheless, at noon, ate of sourcrout and peas, and a small portion of the hog's stomach and its contents. In the afternoon, vomiting again occurred. For two days he had passed nothing from his bowels, and but little urine. In the evening he was forced to take to his bed. On the 22d, Dr. Wolfshofer was called to him. He discovered paralysis of the eyelids, vertigo, slightly coated tongue, hoarseness of voice, intense thirst and great exhaustion. An emetic of eight grains of sulphate of zinc was administered, but did not operate. In the afternoon in addition to the foregoing symptoms, there were great anxiety and dyspnœa, the voice became constantly more hoarse, and during the last three or four hours of his life, the power of speech was entirely lost. His intellect remained, however, undisturbed, and he wrote upon a slate his wants. The difficulty of breathing disappeared a short time before death, which occurred on the evening of April 22.

"2. The wife of the peasant, 36 years old, in good health previously, complained on the 16th of April, of lassitude and occasional pains in the stomach. On the afternoon of the 19th, whilst at work in the vineyard, she experienced great thirst, and on her way home in the evening, she was attacked with vomiting, as also, in the forenoon of the next day, in the vineyard. At noon, as on the 19th, she ate beans and salted and smoked pork. In defiance of her extreme lassitude, she continued at work in the vineyard. On the evening of the 21st, she was obliged, however, to take to her bed. Dr. W. saw her on the morning of the 21st. He found her laboring under paralysis of the eyelids, dilated pupils, some hoarseness of voice and great thirst. The mucous membrane of the fauces was reddened, there was difficulty of swallowing, and a constrictive pain in the region of the œsophagus, directly over the sternum. There was some tumefaction of the stomach, which was sensible to pressure. The bowels had been constipated for three days; the urinary secretion was suppressed; the pulse frequent and weak. A dose of castor oil was given, which produced on the next morning colored stools, and a discharge of urine. The other symptoms were increased, the eyelids could no longer be unclosed, the iris was insensible, the voice scarcely audible, the dysphagia was extreme, the pulse was weak but not frequent, the skin of the extremities was cool. The patient complained of a sense of anxiety. The respiratory murmur was feeble, and here and there mucous râles were heard. The patient expired during the night of the 23d. A short time before her death the difficulty of respiration had disappeared.

"3d. A servant girl, 28 years old, of previous robust health, after partaking of half a liver pudding, on the afternoon of the 19th of April, experienced, whilst working in the vineyard, intense thirst, in consequence of which she drank very copiously of water; she was without appetite, and had pains in the abdomen, but neither vomiting nor diarrhea. On the following day whilst in the vineyard, she vomited once. On the 21st, after partaking of sour beans, she was attacked with repeated vomiting and abdominal pains. Towards evening she was forced to lie down. There had been no evacuation from the bowels or bladder for four days. Pain of the stomach upon pressure. A dose of castor oil was followed by a stool and discharge of urine. On the 22d there occurred repeated vomiting of a yellow fluid, and portions of the peas and beans the patient had eaten. The patient now experienced vertigo, her face became deeply flushed, her pupils were dilated, but without paralysis of the eyelids, the tongue was dry and somewhat coated, there was slight redness of the fauces, and dysphagia without pain. The abdomen was soft, sensible, and tumid. The pulse tolerably full and contracted. On the 25th, a large dose of calomel was administered, and brought away greenish stools, mixed with blood and slime, and attended with some degree of straining. Partial paralysis of the eyelids now set in, with dilation and immobility of the pupils, contraction of the features, a dry hoarse voice, dysphagia, cold extremities, anxiety, and dyspnœa, and the patient expired on the 27th.

"The post-mortem examination, in the cases just detailed, showed the following as the most constant and striking pathological phenomena. Corrugation of the skin, with dryness, and an empty condition of its bloodvessels, and those of the subcutaneous cellular tissue. Congestion of the vessels of the mucous membrane of the air passages and lungs. Hyperæmia of the mucous coat of the intestines, often with ecchymostic effusions. Congestion of the vessels of the kidneys and urinary bladder.

"The course of the disease in the five remaining members of the peasant's family, and in four other individuals not belonging to it, who had eaten of the same blood puddings, was more favorable, and in all terminated without destroying the lives of the patients. In none of these did vomiting occur, but all the leading symptoms observed in the fatal cases were present, to, however, a less intense degree.

"All of the affected individuals had partaken of blood pudding, but the members of the peasant's, of only liver pudding; all were affected with similar symptoms, with, however, various shades of difference. The poisonous property, therefore, had become developed as well in the blood as in the liver puddings, though it would appear that in the latter it was to the greatest extent, inasmuch as in all those persons who had partaken of these, severe disease occurred, while in none of those who had eaten only of the blood puddings, did any dangerous symptoms present themselves.

"The puddings are stated to have had a sour taste, and to have become much softer than they were at first.

"The symptoms observed in the cases, the history of which has been detailed, differ in nothing from those previously recorded as having resulted from sausage poisoning. They may be arranged in two groups, those of reaction and those of paralysis. To the first group appertain the abdominal pains, nausea, vomiting, diarrhea, increased sensibility of the abdomen upon pressure, the hard, full pulse, flushed face, aversion from light. To the second group belong the vertigo, double vision, diminished power of sight, the paralysis, hoarseness of voice running into aphonia, dysphagia amounting sometimes to a total inability to swallow, costiveness, suppression of urinary secretion, cold extremities, and feeble pulse.

"The same interval was observed in all the cases between the consumption of the unwholesome articles and the development of their poisonous effects.

"From the degree of the reaction which occurs in different instances, no conclusions can be drawn as to the severity and danger of the second stage. In the early stage, the symptoms were the same in the cases which terminated fatally as in those of a milder character. It is worthy of especial remark, however, that all the patients who at first suffered from pains in the abdomen and diarrhea, recovered, while in the three patients who died, the cutting pains in the bowels and the vomiting were unattended throughout with diarrhea.

"An examination of the remaining portions of the poisonous articles showed that some of them had become very soft, and exhaled a decidedly sourish and unpleasant smell. This change was not as evident in some portions as in others, which is an additional explanation of the reason why that all who partook of them were not equally affected."—*Austrian Jour. of Prac. Med. and Am. Jour. Med. Sc.*

